# Synthesis, X-ray diffraction and Hirshfeld surface analysis of two new hybrid dihydrate compounds: (C_6_H_22_N_4_)[SnCl_6_]Cl_2_·2H_2_O and (C_8_H_24_N_4_)[SnCl_6_]Cl_2_·2H_2_O

**DOI:** 10.1107/S2056989018001044

**Published:** 2018-01-26

**Authors:** Rafika Bouchene, Zohir Lecheheb, Ratiba Belhouas, Sofiane Bouacida

**Affiliations:** a Département Sciences de la Matière, Faculté des Sciences Exactes et Sciences de la Nature et de la Vie, Université Oum El Bouaghi 04000, Algeria; b Unité de Recherche de Chimie de l’Environnement et Moléculaire Structurale, CHEMS, Faculté des Sciences Exactes, Université des Frères Mentouri Constantine, 25000, Algeria

**Keywords:** crystal structure, stannate complex, hybrid compound, polyamine, Hirshfeld surface analysis

## Abstract

Two new organic–inorganic hybrid compounds, tri­ethyl­ene­tetra­ammonium hexa­chlorido­stannate (IV) dichloride dihydrate, (C_6_H_22_N_4_)[SnCl_6_]Cl_2_·2H_2_O, (I), and 1,4-bis­(2-ammonio­eth­yl)piperazin-1,4-diium hexa­chlorido­stannate (IV) dichloride dihydrate, (C_8_H_24_N_4_)[SnCl_6_]Cl_2_·2H_2_O, (II), have been synthesized from the same starting materials. In each case both the cations and anions are located about inversion centers. Their crystal structures exhibits alternating inorganic and organic stacking sheets in (I) and layers in (II), with Cl^−^ ions and water mol­ecules occupying the space in between. The cohesion of the three-dimensional frameworks are governed by N—H⋯Cl, N—H⋯O, C—H⋯Cl and O—H⋯Cl hydrogen bonds. Hirshfeld surface analysis of both crystal structures indicates that the H⋯Cl/Cl⋯H contacts exert an important influence on the stabilization of the packing.

## Abstract 2

Two new organic–inorganic hybrid compounds have been synthesized from the same starting materials. Their crystal structures exhibits alternating inorganic and organic stacking sheets or layers in (II), with Cl^−^ ions and water mol­ecules occupying the space in between.

## Chemical context   

The introduction of organic components into inorganic systems, to form organic–inorganic hybrid materials, has attracted considerable attention since one would expect new properties that are absent in either of their building blocks (Boopathi *et al.*, 2017 ▸; Newman *et al.*, 1989 ▸; Chun & Jung, 2009 ▸). Moreover, halogenostannate hybrid compounds containing protonated amine cations have received considerable attention thanks to their inter­esting physical and chemical properties, such as magnetic, electroluminescence, photoluminescence and conductivity, which could lead to technological innovations (Aruta *et al.*, 2005 ▸; Chouaib *et al.*, 2015 ▸; Papavassiliou *et al.*, 1999 ▸; Yin & Yo, 1998 ▸). Their structures are generally characterized by isolated or connected chains or clusters of *MX*_6_ octa­hedra separated by the cations.

In this category of materials, the organic moieties, balancing the negative charge on the inorganic parts, usually act as structure-directing agents and greatly affect the structure and the dimensionality of the supra­molecular framework (Díaz *et al.*, 2006 ▸; Hannon *et al.*, 2002 ▸). Furthermore, the experimental conditions employed, such as the solvent, temperature and crystallization method, can also have an important impact on the structure of the final assembly.

As an extension of our previous studies on hybrid N-containing organic halogenometalate materials (Bouacida *et al.*, 2007 ▸, 2009 ▸; Bouchene *et al.*, 2014 ▸), a flexible aliphatic amino template, tri­ethyl­ene­tetra­amine (TETA), was reacted with SnCl_2_ in HCl-acidified aqueous solution. By controlling the temperature, two new organic–inorganic hybrid compounds, tri­ethyl­ene­tetra­ammonium hexa­chlorido­stannate(IV) dichloride dihydrate, (C_6_H_22_N_4_)[SnCl_6_]Cl_2_·2H_2_O (I), and 1,4-bis­(2-ammonio­eth­yl)piperazin-1,4-ium, hexa­chlorido­stannate (IV) dichloride dihydrate, (C_8_H_24_N_4_)[SnCl_6_]Cl_2_·2H_2_O (II), were obtained.
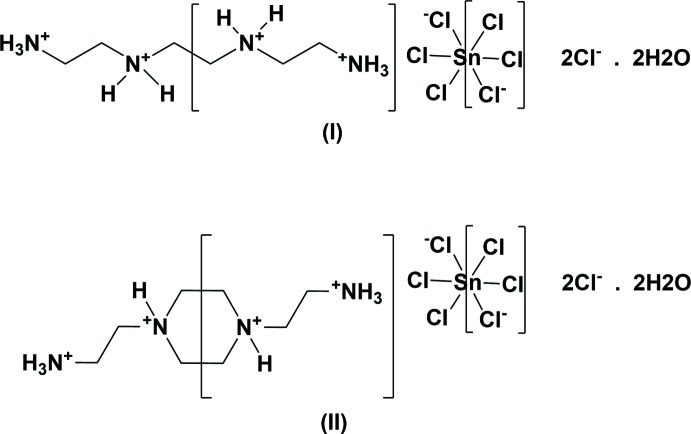


Commercial tri­ethyl­ene­tetra­mine is a mixture of linear TETA (typically 60%) and other branched or cyclic TETA, with close boiling points, such as tris-(2-amino­eth­yl)amine), 1,4-bis­(2-amino­eth­yl)piperazine, (Bis AEP), and *N*-[(2-amino­eth­yl)2-amino­eth­yl]piperazine). Piperazine derivatives are relatively more volatile than the corresponding linear polyethyl­ene amines (Hutchinson *et al.*, 1945 ▸).

The syntheses of (I) and (II) were carried out with the same starting materials but under different reaction temperatures [343 K for (I) and room temperature for (II)]. Surprinsingly, compound (II) was obtained from the reaction of cyclic 1,4-bis­(2-amino­eth­yl)piperazine mol­ecules with SnCl_2_ salt. Under very mild reaction conditions, we believe that (Bis AEP) is present as an impurity in commercial TETA based on the fact that rearrangement reactions of aliphatic chelating polyamines require high pressure and temperature (Liu *et al.*, 2015 ▸). Similar undesired reactions have occurred with the same organic cation (Cukrowski *et al.*, 2012 ▸; Junk & Smith, 2005 ▸; Jiang *et al.*, 2009 ▸; Ye *et al.*, 2002 ▸).

## Structural commentary   

**Figure 1:**
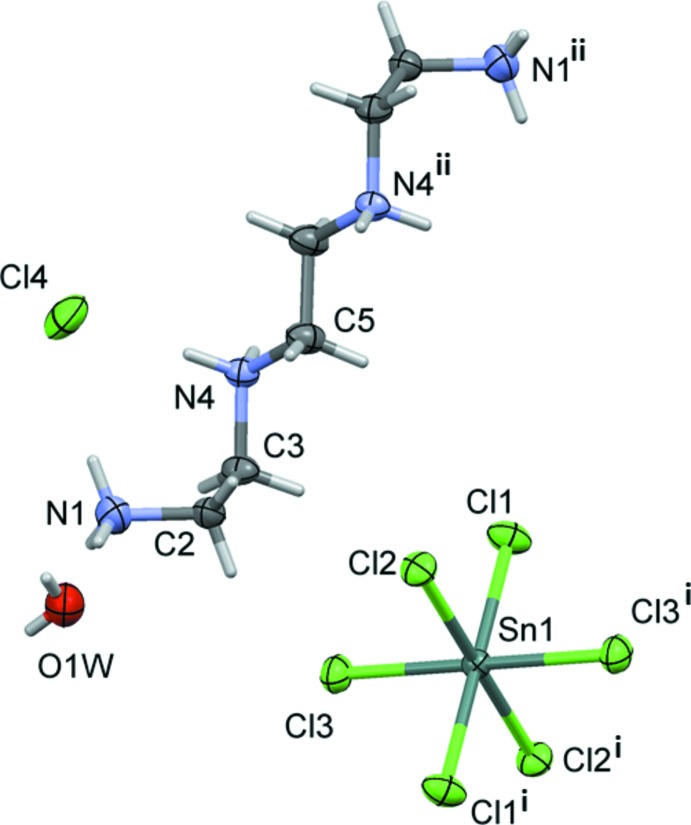
The mol­ecular structure of compound (I), with the atom-numbering scheme for the asymmetric unit. Displacement ellipsoids are drawn at the 50% probability level. Only one Cl^−^ anion and one water mol­ecule are shown. [Symmetry codes: (i) −*x* + 1, −*y* + 2, −*z* + 1; (ii) −*x* + 1, −*y* + 1, −*z* + 1.]

The asymmetric unit of (I) consists of one half of a [TETA]^4+^ cation, one half of an inorganic [SnCl_6_]^2- di^anion, one Cl^−^ ion and one mol­ecule of water (Fig. 1 ▸). The [TETA]^4+^ cation is located about a center of symmetry situated at the middle of the central –CH_2_—CH_2_– bond. The hexa­chlorido­stannate(IV) dianion [SnCl_6_]^2−^, lying on a centre of inversion, exhibits a nearly perfect octa­hedral coordination sphere with Sn—Cl bond lengths ranging from 2.4114 (6) to 2.4469 (6) Å and Cl—Sn—Cl bond angles between 88.94 (2) and 91.06 (2)°.

**Figure 2:**
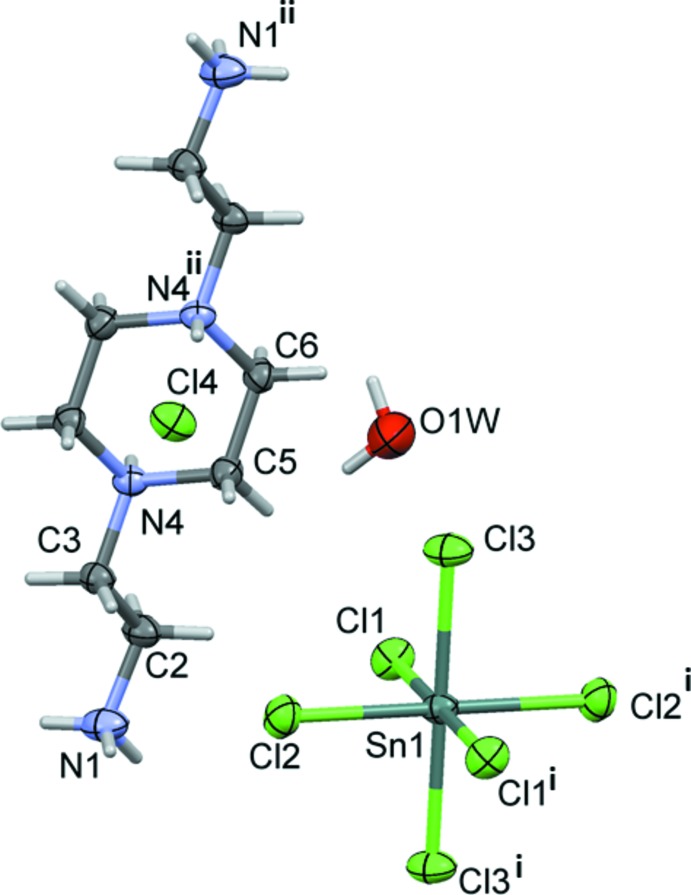
The mol­ecular structure of compound (II), with the atom-numbering scheme for the asymmetric unit. Displacement ellipsoids are drawn at the 50% probability level. Only one Cl^−^ anion and one water mol­ecule are shown. [Symmetry codes: (i) −*x* + 1, −*y* + 1, −*z*; (ii) −*x* + 1, −*y* + 1, −*z* + 1.]

The asymmetric unit of compound (II) contains one half of a [Bis AEP]^4+^ cation, one independent mol­ecule of water, one Cl^−^ ion and half of an [SnCl_6_]^2−^dianion lying on a centre of inversion (Fig. 2 ▸). The [Bis AEP]^4+^ cation is also located about a center of symmetry situated at the center of the piperazin-1,4-diium ring. The nearly perfect octa­hedral coordination around the Sn^IV^ atom is characterized by Sn—Cl bond lengths varying from 2.4265 (6) to 2.4331 (6) Å and Cl—Sn—Cl bond angles ranging from 88.55 (2) to 91.45 (2)° for the *cis* angles [180° for *trans* angles]. The organic part is totally protonated and the piperazinium portion adopts a chair conformation, with both ammonio­ethyl groups being in equatorial positions.

## Supra­molecular features   

**Figure 3:**
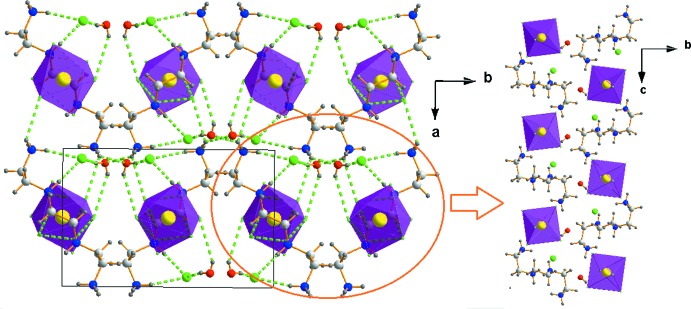
Projection of the crystal packing of (I) wit dashed lines representing hydrogen bonds.

The crystal structure of (I) has an arrangement that can be described as alternating organic [TETA]^4+^ and inorganic [SnCl_6_]^2−^ sheets extending along the *a*-axis direction. The organic cations in adjacent chains are oriented in opposite directions, forming anti­parallel sheets. The isolated chloride ions Cl^−^ and the water mol­ecules are located in the otherwise empty space between the sheets (Fig. 3 ▸).

The crystal packing of (I) is supported by N—H⋯Cl, N—H⋯O*W* and C—H⋯Cl hydrogen-bonding inter­actions (Table 1 ▸). The NH_3_^+^ group as well as the NH_2_^+^ group of [TETA]^4+^ act as hydrogen-bond donors. The *D*⋯*A* distances for the NH_3_^+^ group range from 2.980 (4) to 3.255 (3) Å, while *D*⋯*A* distances of 3.026 (2) to 3.452 (2) Å are found for the NH^2+^ group. The water mol­ecules play an important role in stabilizing the crystal packing of (I) because of their strong ability to form hydrogen bonds with both hydrogen-bond donors and acceptors. By acting as hydrogen-bond donors, they bridge isolated Cl^−^ anions and [SnCl_6_]^2−^ dianions *via* O1*W*—H1*W*⋯Cl4 and O1*W–*-H2*W*⋯Cl2 hydrogen bonds with a H⋯Cl distances of 2.60 (5) and 2.82 (5) Å, respectively. Additionally, by playing the role of acceptors, the water mol­ecules link the inorganic moieties with the organic cations through N1^+^—H1*B*⋯O1*W* and N1^+^—H1*C*⋯O1*W* charge-assisted hydrogen bonds with H⋯O distances of 2.09 and 2.25 Å, respectively.

**Figure 4:**
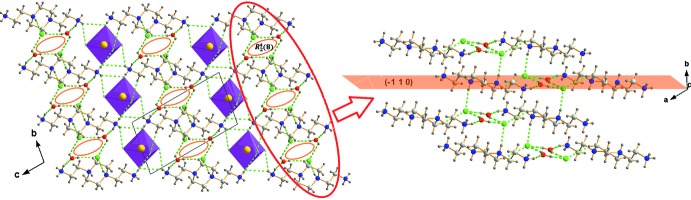
Detail of the hydrogen-bonding inter­actions in the crystal structure of (II). Hydrogen bonds are shown as green dashed lines.

In (II), the isolated chloride ions, located between the [Bis AEP]^4+^ cations, are joined to their adjacent water mol­ecules through strong O*W*—H⋯Cl hydrogen bonds, leading to a hydrogen-bonding pattern with a 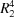
(8) ring motif. The resulting rings, comprising N1^+^—H1*B*⋯O1*W* and C6—H5*B*⋯Cl4 hydrogen bonds, promote the formation of sheets of cations aligned parallel to the (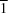 1 0) plane (Table 2 ▸, Fig. 4 ▸). These sheets are linked to each other by charge-assisted iminium-N4^+^—H4⋯Cl4 hydrogen bonds, leading to the formation of organic layers parallel to the *ab* plane. The inorganic layers are built up from isolated [SnCl_6_]^2−^ octa­hedra and alternate with the organic planes along the *c*-axis direction. Each anion is hydrogen bonded to adjacent organic cations through atoms N1 and C2 acting as donors of N—H⋯Cl and C—H⋯Cl hydrogen bonds with N⋯Cl distances varying from 3.343 (2) to 3.431 (2) Å and the C⋯Cl distances of 3.715 (3) Å.

## Hirshfeld surface analysis   

**Figure 5:**
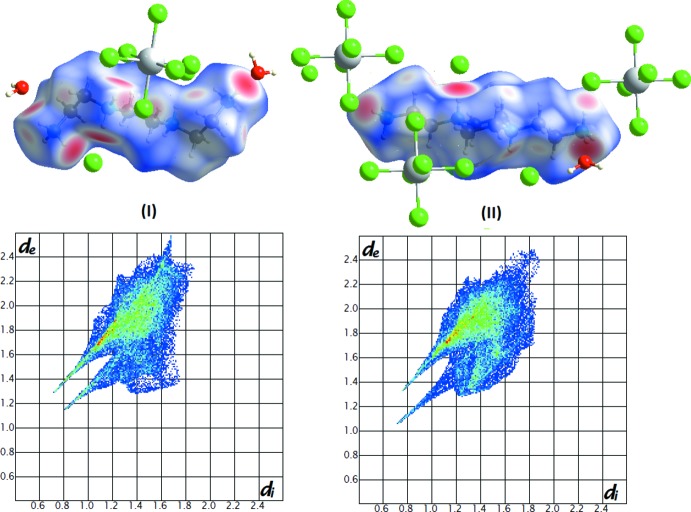
A view of the Hirshfeld surface mapped over *d*_norm_ and two-dimensional fingerprint plots for compounds (I) and (II).

The inter­molecular inter­actions of the obtained structures have been qu­anti­fied using Hirshfeld surface analysis. *CrystalExplorer* software (Wolff *et al.*, 2007 ▸) was used to generate the Hirshfeld surface and two-dimensional fingerprint (FP) plots. The analysis of the inter­molecular inter­actions through the mapping of *d*_norm_ is permitted by the contact distances *d*_i_ and *d*_e_ from the Hirshfeld surface to the nearest atom inside and outside, respectively. The surface mapped over *d*_norm_ displays red spots that correspond to contacts shorter than the sum of the van der Waals radii, as shown in Fig. 5 ▸.

In compounds (I) and (II), isolated Cl atoms act as potential acceptors for hydrogen bonds; this explains why the greatest contribution to the Hirshfeld surface [65.9% for (I) and 59.8% for (II)] is from the H⋯Cl/Cl⋯H contacts. As expected in organic compounds, the H⋯H contacts are the second important contribution, *i.e*. 24.8% and 30.7% for (I) and (II), respectively. It is evident that van der Waals forces exert an important influence on the stabilization of the packing in the crystal structure. Since both compounds are hydrated, the fingerprint plots also show H⋯O/O⋯H contacts that contribute less to the Hirshfeld surfaces, making contributions of 9.3 and 9.5%, respectively.

## Database survey   

A search of the Cambridge Structural Database (Version 5.38, update May 2017; Groom *et al.*, 2016 ▸) revealed no obvious analogues of (I) and (II) in the crystallographic literature. The structures of related hydrated salts with the same cations, *i.e.* tri­ethyl­ene­tetra­minium bis­(sulfate) monohydrate, (C_6_H_22_N_4_)SO_4_·H_2_O (III), and bis­(2-ammonio­eth­yl)piperazin-1,4-ium tetra­perchlorate tetra­hydrate, (C_8_H_24_N_4_)_4_ClO_4_·4H_2_O (IV), have been reported (Fu *et al.*, 2005 ▸; Ye *et al.*, 2002 ▸). Compound (III) was obtained indirectly by a hydro­thermal synthesis using a mixture of ferric sulfate nona­hydrate and tri­ethyl­ene­tetra­amine. The ionic product (IV) was also an unexpected product from the reaction between tri­ethyl­ene­tetra­mine and perchloric acid. The cationic portion of the structure adopts a chair conformation and the experimental distances are close to those for the neutral ligand.

## Synthesis and crystallization   

All chemicals were used without further purification*.* A solution of an aqueous mixture of tin chloride (SnCl_2_) and tetra­ethyl­ene­tetra­amine in an HCl-acidified medium with a stoichiometric ratio of 1:1 was refluxed for one h at 343 K for (I) and room temperature for (II). After two weeks of slow solvent evaporation, single crystals suitable for X-ray analysis were obtained.

## Refinement   

Crystal data, data collection and structure refinement details are summarized in Table 3 ▸. Approximate positions for all H atoms were first obtained from difference-Fourier maps. H atoms were then placed idealized positions and refined using the riding-atom approximation: C—H = 0.93 Å and N—H = 0.86 Å, with *U*_iso_(H) = 1.2*U*_eq_(C,N). H atoms of the water mol­ecule were located in a difference-Fourier map and refined with *U*_iso_(H) = 1.5*U*_eq_(O).

## Supplementary Material

Crystal structure: contains datablock(s) global, I, II. DOI: 10.1107/S2056989018001044/tx2003sup1.cif

CCDC references: 1817660, 1817659

Additional supporting information:  crystallographic information; 3D view; checkCIF report

## Figures and Tables

**Table 1:** Hydrogen-bond geometry (Å, °) for (I)

*D*—H⋯*A*	*D*—H	H⋯*A*	*D*⋯*A*	*D*—H⋯*A*
N1—H1*A*⋯Cl4	0.89	2.30	3.172 (2)	167
N1—H1*B*⋯O1*W*	0.89	2.09	2.980 (4)	179
N1—H1*C*⋯Cl1^i^	0.89	2.75	3.255 (3)	117
N1—H1*C*⋯O1*W* ^ii^	0.89	2.25	3.037 (4)	147
O1*W*—H1*W*⋯Cl4^iii^	0.75 (4)	2.60 (5)	3.281 (3)	151 (4)
O1*W*—H2*W*⋯Cl2^i^	0.72 (5)	2.82 (5)	3.422 (3)	144 (4)
N4—H4*A*⋯Cl2^ii^	0.90	2.50	3.2225 (19)	138
N4—H4*A*⋯Cl1^iv^	0.90	2.75	3.452 (2)	136
N4—H4*B*⋯Cl4	0.90	2.13	3.026 (2)	173
C5—H5*B*⋯Cl1^v^	0.97	2.76	3.445 (3)	128

Symmetry codes: (i) 
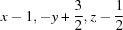
; (ii) 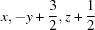; (iii) 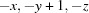; (iv) 
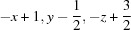
; (v) 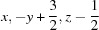.

**Table 2:** Hydrogen-bond geometry (Å, °) for (II)

*D*—H⋯*A*	*D*—H	H⋯*A*	*D*⋯*A*	*D*—H⋯*A*
N1—H1*A*⋯Cl3^i^	0.89	2.71	3.397 (2)	134
N1—H1*A*⋯Cl2^ii^	0.89	2.81	3.431 (2)	128
N1—H1*B*⋯Cl1^iii^	0.89	2.47	3.343 (2)	167
N1—H1*C*⋯O1*W* ^i^	0.89	1.92	2.769 (4)	158
O1*W*—H1*W*⋯Cl4^iv^	0.83 (2)	2.30 (3)	3.079 (3)	158 (6)
O1*W*—H2*W*⋯Cl4	0.83 (4)	2.67 (5)	3.246 (3)	128 (5)
N4—H4⋯Cl4	0.85 (4)	2.24 (4)	3.073 (2)	164 (3)
C2—H2*B*⋯Cl1	0.97	2.79	3.715 (3)	160
C6—H6*A*⋯Cl4^v^	0.97	2.70	3.506 (3)	141

Symmetry codes: (i) 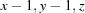; (ii) 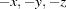; (iii) 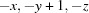; (iv) 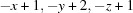; (v) 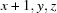.

**Table 3:** Experimental details

	(I)	(II)
Crystal data		
Chemical formula	(C_6_H_22_N_4_)[SnCl_6_]Cl_2_·2H_2_O	(C_8_H_24_N_4_)[SnCl_6_]Cl_2_·2H_2_O
*M*_r_	588.62	614.65
Crystal system, space group	Monoclinic, *P*2_1_/*c*	Triclinic, *P* 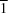
Temperature (K)	295	295
*a*, *b*, *c* (Å)	8.7573 (2), 12.8372 (3), 9.7103 (2)	7.0856 (2), 7.3269 (2), 12.1624 (4)
α, β, γ (°)	90, 107.265 (1), 90	93.614 (2), 101.357 (1), 117.021 (2)
*V* (Å^3^)	1042.44 (4)	543.01 (3)
*Z*	2	1
Radiation type	Mo *K*α	Mo *K*α
μ (mm^−1^)	2.26	2.17
Crystal size (mm)	0.12 × 0.04 × 0.03	0.13 × 0.12 × 0.11

Data collection		
Diffractometer	Nonius KappaCCD	Nonius KappaCCD
Absorption correction	Multi-scan (*SADABS*; Krause *et al.*, 2015 ▸)	Multi-scan (*SADABS*; Krause *et al.*, 2015 ▸)
*T*_min_, *T*_max_	0.665, 0.871	0.745, 0.893
No. of measured, independent and observed [*I* > 2σ(*I*)] reflections	4666, 2394, 2133	4329, 2494, 2319
*R*_int_	0.016	0.014
(sin θ/λ)_max_ (Å^−1^)	0.650	0.650

Refinement		
*R*[*F* ^2^ > 2σ(*F* ^2^)], *wR*(*F* ^2^), *S*	0.027, 0.065, 1.17	0.025, 0.064, 1.13
No. of reflections	2394	2494
No. of parameters	104	117
No. of restraints	0	2
H-atom treatment	H atoms treated by a mixture of independent and constrained refinement	H atoms treated by a mixture of independent and constrained refinement
Δρ_max_, Δρ_min_ (e Å^−3^)	0.84, −0.75	0.61, −0.65

Computer programs: *COLLECT* (Nonius, 1998 ▸), *DENZO* and *SCALEPACK* (Otwinowski & Minor, 1997 ▸), *SHELXS97* and *SHELXL97* (Sheldrick, 2008 ▸), *ORTEP-3 for Windows* and *WinGX* (Farrugia, 2012 ▸) and *DIAMOND* (Brandenburg & Berndt, 2001 ▸).

## supplementary crystallographic information

### Triethylenetetraammonium hexachloridostannate(IV) dichloride dihydrate (I) Crystal data

**Table:**

(C_6_H_22_N_4_)[SnCl_6_]Cl_2_·2H_2_O	*F*(000) = 584
*M**_r_* = 588.62	*D*_x_ = 1.875 Mg m^−^^3^
Monoclinic, *P*2_1_/*c*	Mo *K*α radiation, λ = 0.71073 Å
Hall symbol: -P 2ybc	Cell parameters from 2499 reflections
*a* = 8.7573 (2) Å	θ = 2.9–27.5°
*b* = 12.8372 (3) Å	µ = 2.26 mm^−^^1^
*c* = 9.7103 (2) Å	*T* = 295 K
β = 107.265 (1)°	Needle, colorless
*V* = 1042.44 (4) Å^3^	0.12 × 0.04 × 0.03 mm
*Z* = 2	

### Triethylenetetraammonium hexachloridostannate(IV) dichloride dihydrate (I) Data collection

**Table:**

Nonius KappaCCD diffractometer	2394 independent reflections
Radiation source: Enraf Nonius FR590	2133 reflections with *I* > 2σ(*I*)
Graphite monochromator	*R*_int_ = 0.016
Detector resolution: 9 pixels mm^-1^	θ_max_ = 27.5°, θ_min_ = 3.2°
CCD rotation images, thick slices scans	*h* = −11→11
Absorption correction: multi-scan (SADABS; Krause et al., 2015)	*k* = −16→16
*T*_min_ = 0.665, *T*_max_ = 0.871	*l* = −12→12
4666 measured reflections	

### Triethylenetetraammonium hexachloridostannate(IV) dichloride dihydrate (I) Refinement

**Table:**

Refinement on *F*^2^	Primary atom site location: structure-invariant direct methods
Least-squares matrix: full	Secondary atom site location: difference Fourier map
*R*[*F*^2^ > 2σ(*F*^2^)] = 0.027	Hydrogen site location: inferred from neighbouring sites
*wR*(*F*^2^) = 0.065	H atoms treated by a mixture of independent and constrained refinement
*S* = 1.17	*w* = 1/[σ^2^(*F*_o_^2^) + (0.023*P*)^2^ + 0.5523*P*] where *P* = (*F*_o_^2^ + 2*F*_c_^2^)/3
2394 reflections	(Δ/σ)_max_ = 0.003
104 parameters	Δρ_max_ = 0.84 e Å^−^^3^
0 restraints	Δρ_min_ = −0.75 e Å^−^^3^

### Triethylenetetraammonium hexachloridostannate(IV) dichloride dihydrate (I) Special details

**Table:**

Geometry. All esds (except the esd in the dihedral angle between two l.s. planes) are estimated using the full covariance matrix. The cell esds are taken into account individually in the estimation of esds in distances, angles and torsion angles; correlations between esds in cell parameters are only used when they are defined by crystal symmetry. An approximate (isotropic) treatment of cell esds is used for estimating esds involving l.s. planes.
Refinement. Refinement of F^2^ against ALL reflections. The weighted R-factor wR and goodness of fit S are based on F^2^, conventional R-factors R are based on F, with F set to zero for negative F^2^. The threshold expression of F^2^ > 2sigma(F^2^) is used only for calculating R-factors(gt) etc. and is not relevant to the choice of reflections for refinement. R-factors based on F^2^ are statistically about twice as large as those based on F, and R- factors based on ALL data will be even larger.

### Triethylenetetraammonium hexachloridostannate(IV) dichloride dihydrate (I) Fractional atomic coordinates and isotropic or equivalent isotropic displacement parameters (Å^2^)

**Table:**

	*x*	*y*	*z*	*U*_iso_*/*U*_eq_	
Sn1	0.5	1	0.5	0.02085 (8)	
Cl3	0.24253 (8)	0.95074 (5)	0.52198 (7)	0.03506 (15)	
Cl1	0.63022 (8)	0.88957 (5)	0.70418 (7)	0.03609 (16)	
Cl2	0.49558 (7)	0.85219 (5)	0.33975 (6)	0.03185 (15)	
N4	0.3155 (2)	0.56699 (15)	0.5183 (2)	0.0258 (4)	
H4A	0.3399	0.5572	0.6141	0.031*	
H4B	0.2434	0.5182	0.4751	0.031*	
C5	0.4621 (3)	0.5521 (2)	0.4738 (3)	0.0339 (6)	
H5A	0.5376	0.6075	0.5136	0.041*	
H5B	0.4352	0.5552	0.3695	0.041*	
O1W	−0.1031 (3)	0.69980 (19)	−0.0541 (3)	0.0483 (6)	
H1W	−0.107 (5)	0.657 (3)	−0.108 (5)	0.072*	
H2W	−0.183 (6)	0.713 (3)	−0.054 (5)	0.072*	
Cl4	0.07323 (11)	0.41040 (6)	0.34986 (8)	0.0520 (2)	
N1	0.0113 (3)	0.64761 (18)	0.2588 (3)	0.0431 (6)	
H1A	0.0167	0.5788	0.2701	0.065*	
H1B	−0.0239	0.6629	0.1653	0.065*	
H1C	−0.0557	0.6737	0.3032	0.065*	
C2	0.1726 (3)	0.69341 (19)	0.3220 (3)	0.0287 (5)	
H2A	0.1665	0.7681	0.3058	0.034*	
H2B	0.2446	0.6649	0.2726	0.034*	
C3	0.2409 (3)	0.67290 (18)	0.4817 (3)	0.0286 (5)	
H3A	0.321	0.7254	0.523	0.034*	
H3B	0.1561	0.6804	0.5265	0.034*	

### Triethylenetetraammonium hexachloridostannate(IV) dichloride dihydrate (I) Atomic displacement parameters (Å^2^)

**Table:**

	*U*^11^	*U*^22^	*U*^33^	*U*^12^	*U*^13^	*U*^23^
Sn1	0.02192 (13)	0.02125 (13)	0.01970 (13)	−0.00032 (7)	0.00668 (9)	0.00003 (7)
Cl3	0.0287 (3)	0.0335 (3)	0.0469 (4)	−0.0049 (2)	0.0173 (3)	−0.0010 (3)
Cl1	0.0401 (4)	0.0393 (3)	0.0293 (3)	0.0120 (3)	0.0110 (3)	0.0106 (3)
Cl2	0.0358 (3)	0.0287 (3)	0.0298 (3)	0.0001 (2)	0.0079 (3)	−0.0077 (2)
N4	0.0284 (10)	0.0262 (10)	0.0225 (9)	0.0025 (8)	0.0069 (8)	0.0012 (8)
C5	0.0337 (13)	0.0328 (14)	0.0399 (14)	0.0088 (11)	0.0181 (11)	0.0088 (11)
O1W	0.0394 (12)	0.0462 (13)	0.0578 (14)	−0.0046 (10)	0.0122 (11)	−0.0039 (10)
Cl4	0.0690 (5)	0.0346 (4)	0.0455 (4)	−0.0195 (4)	0.0064 (4)	−0.0031 (3)
N1	0.0338 (13)	0.0401 (13)	0.0473 (14)	−0.0031 (10)	−0.0002 (11)	0.0069 (11)
C2	0.0270 (12)	0.0276 (12)	0.0308 (12)	0.0023 (9)	0.0073 (10)	0.0040 (10)
C3	0.0335 (13)	0.0245 (11)	0.0281 (12)	0.0051 (10)	0.0094 (10)	−0.0020 (9)

### Triethylenetetraammonium hexachloridostannate(IV) dichloride dihydrate (I) Geometric parameters (Å, º)

**Table:**

Sn1—Cl3	2.4114 (6)	C5—H5B	0.97
Sn1—Cl3^i^	2.4114 (6)	O1W—H1W	0.75 (4)
Sn1—Cl1	2.4288 (6)	O1W—H2W	0.72 (4)
Sn1—Cl1^i^	2.4288 (6)	N1—C2	1.484 (3)
Sn1—Cl2	2.4469 (6)	N1—H1A	0.89
Sn1—Cl2^i^	2.4469 (6)	N1—H1B	0.89
N4—C5	1.484 (3)	N1—H1C	0.89
N4—C3	1.505 (3)	C2—C3	1.510 (3)
N4—H4A	0.9	C2—H2A	0.97
N4—H4B	0.9	C2—H2B	0.97
C5—C5^ii^	1.512 (5)	C3—H3A	0.97
C5—H5A	0.97	C3—H3B	0.97
			
Cl3—Sn1—Cl3^i^	180	C5^ii^—C5—H5A	109.6
Cl3—Sn1—Cl1	89.97 (2)	N4—C5—H5B	109.6
Cl3^i^—Sn1—Cl1	90.03 (2)	C5^ii^—C5—H5B	109.6
Cl3—Sn1—Cl1^i^	90.03 (2)	H5A—C5—H5B	108.1
Cl3^i^—Sn1—Cl1^i^	89.97 (2)	H1W—O1W—H2W	109 (5)
Cl1—Sn1—Cl1^i^	180	C2—N1—H1A	109.5
Cl3—Sn1—Cl2	90.81 (2)	C2—N1—H1B	109.5
Cl3^i^—Sn1—Cl2	89.19 (2)	H1A—N1—H1B	109.5
Cl1—Sn1—Cl2	88.94 (2)	C2—N1—H1C	109.5
Cl1^i^—Sn1—Cl2	91.06 (2)	H1A—N1—H1C	109.5
Cl3—Sn1—Cl2^i^	89.19 (2)	H1B—N1—H1C	109.5
Cl3^i^—Sn1—Cl2^i^	90.81 (2)	N1—C2—C3	113.2 (2)
Cl1—Sn1—Cl2^i^	91.06 (2)	N1—C2—H2A	108.9
Cl1^i^—Sn1—Cl2^i^	88.94 (2)	C3—C2—H2A	108.9
Cl2—Sn1—Cl2^i^	180	N1—C2—H2B	108.9
C5—N4—C3	113.62 (19)	C3—C2—H2B	108.9
C5—N4—H4A	108.8	H2A—C2—H2B	107.8
C3—N4—H4A	108.8	N4—C3—C2	114.31 (19)
C5—N4—H4B	108.8	N4—C3—H3A	108.7
C3—N4—H4B	108.8	C2—C3—H3A	108.7
H4A—N4—H4B	107.7	N4—C3—H3B	108.7
N4—C5—C5^ii^	110.3 (3)	C2—C3—H3B	108.7
N4—C5—H5A	109.6	H3A—C3—H3B	107.6
			
C3—N4—C5—C5^ii^	174.8 (3)	N1—C2—C3—N4	81.1 (3)
C5—N4—C3—C2	66.8 (3)		

Symmetry codes: (i) −*x*+1, −*y*+2, −*z*+1; (ii) −*x*+1, −*y*+1, −*z*+1.

### Triethylenetetraammonium hexachloridostannate(IV) dichloride dihydrate (I) Hydrogen-bond geometry (Å, º)

**Table:**

*D*—H···*A*	*D*—H	H···*A*	*D*···*A*	*D*—H···*A*
N1—H1*A*···Cl4	0.89	2.30	3.172 (2)	167
N1—H1*B*···O1*W*	0.89	2.09	2.980 (4)	179
N1—H1*C*···Cl1^iii^	0.89	2.75	3.255 (3)	117
N1—H1*C*···O1*W*^iv^	0.89	2.25	3.037 (4)	147
O1*W*—H1*W*···Cl4^v^	0.75 (4)	2.60 (5)	3.281 (3)	151 (4)
O1*W*—H2*W*···Cl2^iii^	0.72 (5)	2.82 (5)	3.422 (3)	144 (4)
N4—H4*A*···Cl2^iv^	0.90	2.50	3.2225 (19)	138
N4—H4*A*···Cl1^vi^	0.90	2.75	3.452 (2)	136
N4—H4*B*···Cl4	0.90	2.13	3.026 (2)	173
C5—H5*B*···Cl1^vii^	0.97	2.76	3.445 (3)	128

Symmetry codes: (iii) *x*−1, −*y*+3/2, *z*−1/2; (iv) *x*, −*y*+3/2, *z*+1/2; (v) −*x*, −*y*+1, −*z*; (vi) −*x*+1, *y*−1/2, −*z*+3/2; (vii) *x*, −*y*+3/2, *z*−1/2.

### 1,4-Bis(2-ammonioethyl)piperazin-1,4-diium hexachloridostannate(IV) dichloride dihydrate (II) Crystal data

**Table:**

(C_8_H_24_N_4_)[SnCl_6_]Cl_2_·2H_2_O	*Z* = 1
*M**_r_* = 614.65	*F*(000) = 306
Triclinic, *P*1	*D*_x_ = 1.88 Mg m^−^^3^
Hall symbol: -P 1	Mo *K*α radiation, λ = 0.71073 Å
*a* = 7.0856 (2) Å	Cell parameters from 5436 reflections
*b* = 7.3269 (2) Å	θ = 2.9–27.5°
*c* = 12.1624 (4) Å	µ = 2.17 mm^−^^1^
α = 93.614 (2)°	*T* = 295 K
β = 101.357 (1)°	Cube, colorless
γ = 117.021 (2)°	0.13 × 0.12 × 0.11 mm
*V* = 543.01 (3) Å^3^	

### 1,4-Bis(2-ammonioethyl)piperazin-1,4-diium hexachloridostannate(IV) dichloride dihydrate (II) Data collection

**Table:**

Nonius KappaCCD diffractometer	2494 independent reflections
Radiation source: Enraf Nonius FR590	2319 reflections with *I* > 2σ(*I*)
Graphite monochromator	*R*_int_ = 0.014
Detector resolution: 9 pixels mm^-1^	θ_max_ = 27.5°, θ_min_ = 3.2°
CCD rotation images, thick slices scans	*h* = −9→9
Absorption correction: multi-scan (SADABS; Krause et al., 2015)	*k* = −9→9
*T*_min_ = 0.745, *T*_max_ = 0.893	*l* = −15→15
4329 measured reflections	

### 1,4-Bis(2-ammonioethyl)piperazin-1,4-diium hexachloridostannate(IV) dichloride dihydrate (II) Refinement

**Table:**

Refinement on *F*^2^	Secondary atom site location: difference Fourier map
Least-squares matrix: full	Hydrogen site location: inferred from neighbouring sites
*R*[*F*^2^ > 2σ(*F*^2^)] = 0.025	H atoms treated by a mixture of independent and constrained refinement
*wR*(*F*^2^) = 0.064	*w* = 1/[σ^2^(*F*_o_^2^) + (0.025*P*)^2^ + 0.3256*P*] where *P* = (*F*_o_^2^ + 2*F*_c_^2^)/3
*S* = 1.13	(Δ/σ)_max_ < 0.001
2494 reflections	Δρ_max_ = 0.61 e Å^−^^3^
117 parameters	Δρ_min_ = −0.65 e Å^−^^3^
2 restraints	Extinction correction: SHELXL, Fc^*^=kFc[1+0.001xFc^2^λ^3^/sin(2θ)]^-1/4^
Primary atom site location: structure-invariant direct methods	Extinction coefficient: 0.033 (2)

### 1,4-Bis(2-ammonioethyl)piperazin-1,4-diium hexachloridostannate(IV) dichloride dihydrate (II) Special details

**Table:**

Geometry. All esds (except the esd in the dihedral angle between two l.s. planes) are estimated using the full covariance matrix. The cell esds are taken into account individually in the estimation of esds in distances, angles and torsion angles; correlations between esds in cell parameters are only used when they are defined by crystal symmetry. An approximate (isotropic) treatment of cell esds is used for estimating esds involving l.s. planes.
Refinement. Refinement of F^2^ against ALL reflections. The weighted R-factor wR and goodness of fit S are based on F^2^, conventional R-factors R are based on F, with F set to zero for negative F^2^. The threshold expression of F^2^ > 2sigma(F^2^) is used only for calculating R-factors(gt) etc. and is not relevant to the choice of reflections for refinement. R-factors based on F^2^ are statistically about twice as large as those based on F, and R- factors based on ALL data will be even larger.

### 1,4-Bis(2-ammonioethyl)piperazin-1,4-diium hexachloridostannate(IV) dichloride dihydrate (II) Fractional atomic coordinates and isotropic or equivalent isotropic displacement parameters (Å^2^)

**Table:**

	*x*	*y*	*z*	*U*_iso_*/*U*_eq_	
Sn1	0.5	0.5	0	0.02689 (10)	
Cl3	0.78922 (11)	0.66433 (10)	0.17479 (5)	0.04033 (16)	
Cl4	0.17862 (10)	0.75981 (9)	0.44661 (6)	0.03755 (16)	
Cl1	0.30602 (10)	0.66732 (10)	0.06748 (5)	0.03706 (15)	
Cl2	0.31206 (12)	0.19729 (9)	0.08532 (6)	0.04023 (16)	
N4	0.2782 (3)	0.3944 (3)	0.42328 (16)	0.0239 (4)	
H4	0.238 (5)	0.484 (5)	0.440 (2)	0.036*	
C3	0.0936 (4)	0.2192 (3)	0.33497 (19)	0.0269 (5)	
H3A	−0.0176	0.1302	0.3709	0.032*	
H3B	0.1481	0.1361	0.3005	0.032*	
O1W	0.5368 (4)	1.0237 (5)	0.3102 (2)	0.0736 (8)	
H1W	0.579 (9)	1.068 (8)	0.3794 (19)	0.11*	
H2W	0.404 (4)	0.939 (7)	0.297 (5)	0.11*	
N1	−0.1969 (4)	0.1317 (3)	0.16070 (19)	0.0392 (5)	
H1A	−0.1568	0.0426	0.1338	0.059*	
H1B	−0.2451	0.1822	0.1035	0.059*	
H1C	−0.3032	0.0662	0.195	0.059*	
C6	0.6631 (4)	0.6818 (3)	0.4703 (2)	0.0275 (5)	
H6A	0.7909	0.7482	0.4404	0.033*	
H6B	0.6187	0.7849	0.4884	0.033*	
C2	−0.0067 (4)	0.3044 (4)	0.2435 (2)	0.0314 (5)	
H2A	−0.0531	0.3943	0.2786	0.038*	
H2B	0.1021	0.3864	0.2044	0.038*	
C5	0.4795 (4)	0.5058 (4)	0.3805 (2)	0.0284 (5)	
H5A	0.4439	0.561	0.3136	0.034*	
H5B	0.5277	0.4078	0.3582	0.034*	

### 1,4-Bis(2-ammonioethyl)piperazin-1,4-diium hexachloridostannate(IV) dichloride dihydrate (II) Atomic displacement parameters (Å^2^)

**Table:**

	*U*^11^	*U*^22^	*U*^33^	*U*^12^	*U*^13^	*U*^23^
Sn1	0.03243 (14)	0.02481 (13)	0.02285 (14)	0.01475 (10)	0.00329 (9)	0.00328 (8)
Cl3	0.0426 (3)	0.0413 (3)	0.0303 (3)	0.0212 (3)	−0.0060 (3)	−0.0003 (3)
Cl4	0.0368 (3)	0.0320 (3)	0.0449 (4)	0.0191 (3)	0.0078 (3)	0.0000 (3)
Cl1	0.0443 (3)	0.0386 (3)	0.0376 (3)	0.0264 (3)	0.0129 (3)	0.0076 (3)
Cl2	0.0553 (4)	0.0302 (3)	0.0372 (3)	0.0189 (3)	0.0180 (3)	0.0115 (2)
N4	0.0235 (9)	0.0253 (9)	0.0233 (9)	0.0133 (7)	0.0031 (7)	0.0029 (7)
C3	0.0272 (10)	0.0248 (10)	0.0255 (11)	0.0120 (9)	0.0016 (9)	0.0030 (8)
O1W	0.0596 (15)	0.087 (2)	0.0444 (14)	0.0113 (14)	0.0113 (12)	0.0132 (14)
N1	0.0456 (12)	0.0386 (12)	0.0278 (11)	0.0211 (10)	−0.0039 (9)	0.0011 (9)
C6	0.0258 (10)	0.0277 (11)	0.0275 (11)	0.0114 (9)	0.0058 (9)	0.0100 (9)
C2	0.0314 (11)	0.0276 (11)	0.0281 (12)	0.0116 (9)	−0.0007 (9)	0.0035 (9)
C5	0.0271 (11)	0.0342 (12)	0.0242 (11)	0.0146 (9)	0.0070 (9)	0.0072 (9)

### 1,4-Bis(2-ammonioethyl)piperazin-1,4-diium hexachloridostannate(IV) dichloride dihydrate (II) Geometric parameters (Å, º)

**Table:**

Sn1—Cl3	2.4265 (6)	O1W—H2W	0.836 (19)
Sn1—Cl3^i^	2.4265 (6)	N1—C2	1.479 (3)
Sn1—Cl1^i^	2.4319 (6)	N1—H1A	0.89
Sn1—Cl1	2.4319 (6)	N1—H1B	0.89
Sn1—Cl2	2.4331 (6)	N1—H1C	0.89
Sn1—Cl2^i^	2.4331 (6)	C6—N4^ii^	1.500 (3)
N4—C6^ii^	1.500 (3)	C6—C5	1.512 (3)
N4—C5	1.503 (3)	C6—H6A	0.97
N4—C3	1.503 (3)	C6—H6B	0.97
N4—H4	0.85 (3)	C2—H2A	0.97
C3—C2	1.520 (3)	C2—H2B	0.97
C3—H3A	0.97	C5—H5A	0.97
C3—H3B	0.97	C5—H5B	0.97
O1W—H1W	0.826 (19)		
			
Cl3—Sn1—Cl3^i^	180	H3A—C3—H3B	108.1
Cl3—Sn1—Cl1^i^	90.31 (2)	H1W—O1W—H2W	106 (5)
Cl3^i^—Sn1—Cl1^i^	89.69 (2)	C2—N1—H1A	109.5
Cl3—Sn1—Cl1	89.69 (2)	C2—N1—H1B	109.5
Cl3^i^—Sn1—Cl1	90.31 (2)	H1A—N1—H1B	109.5
Cl1^i^—Sn1—Cl1	180.00 (3)	C2—N1—H1C	109.5
Cl3—Sn1—Cl2	90.42 (2)	H1A—N1—H1C	109.5
Cl3^i^—Sn1—Cl2	89.58 (2)	H1B—N1—H1C	109.5
Cl1^i^—Sn1—Cl2	88.55 (2)	N4^ii^—C6—C5	111.57 (18)
Cl1—Sn1—Cl2	91.45 (2)	N4^ii^—C6—H6A	109.3
Cl3—Sn1—Cl2^i^	89.58 (2)	C5—C6—H6A	109.3
Cl3^i^—Sn1—Cl2^i^	90.42 (2)	N4^ii^—C6—H6B	109.3
Cl1^i^—Sn1—Cl2^i^	91.45 (2)	C5—C6—H6B	109.3
Cl1—Sn1—Cl2^i^	88.55 (2)	H6A—C6—H6B	108
Cl2—Sn1—Cl2^i^	180	N1—C2—C3	110.21 (19)
C6^ii^—N4—C5	109.02 (17)	N1—C2—H2A	109.6
C6^ii^—N4—C3	111.27 (17)	C3—C2—H2A	109.6
C5—N4—C3	112.28 (18)	N1—C2—H2B	109.6
C6^ii^—N4—H4	109 (2)	C3—C2—H2B	109.6
C5—N4—H4	107 (2)	H2A—C2—H2B	108.1
C3—N4—H4	109 (2)	N4—C5—C6	111.52 (19)
N4—C3—C2	110.33 (18)	N4—C5—H5A	109.3
N4—C3—H3A	109.6	C6—C5—H5A	109.3
C2—C3—H3A	109.6	N4—C5—H5B	109.3
N4—C3—H3B	109.6	C6—C5—H5B	109.3
C2—C3—H3B	109.6	H5A—C5—H5B	108
			
C6^ii^—N4—C3—C2	163.67 (19)	C6^ii^—N4—C5—C6	−56.4 (3)
C5—N4—C3—C2	−73.8 (2)	C3—N4—C5—C6	179.86 (18)
N4—C3—C2—N1	−176.51 (19)	N4^ii^—C6—C5—N4	57.8 (3)

Symmetry codes: (i) −*x*+1, −*y*+1, −*z*; (ii) −*x*+1, −*y*+1, −*z*+1.

### 1,4-Bis(2-ammonioethyl)piperazin-1,4-diium hexachloridostannate(IV) dichloride dihydrate (II) Hydrogen-bond geometry (Å, º)

**Table:**

*D*—H···*A*	*D*—H	H···*A*	*D*···*A*	*D*—H···*A*
N1—H1*A*···Cl3^iii^	0.89	2.71	3.397 (2)	134
N1—H1*A*···Cl2^iv^	0.89	2.81	3.431 (2)	128
N1—H1*B*···Cl1^v^	0.89	2.47	3.343 (2)	167
N1—H1*C*···O1*W*^iii^	0.89	1.92	2.769 (4)	158
O1*W*—H1*W*···Cl4^vi^	0.83 (2)	2.30 (3)	3.079 (3)	158 (6)
O1*W*—H2*W*···Cl4	0.83 (4)	2.67 (5)	3.246 (3)	128 (5)
N4—H4···Cl4	0.85 (4)	2.24 (4)	3.073 (2)	164 (3)
C2—H2*B*···Cl1	0.97	2.79	3.715 (3)	160
C6—H6*A*···Cl4^vii^	0.97	2.70	3.506 (3)	141

Symmetry codes: (iii) *x*−1, *y*−1, *z*; (iv) −*x*, −*y*, −*z*; (v) −*x*, −*y*+1, −*z*; (vi) −*x*+1, −*y*+2, −*z*+1; (vii) *x*+1, *y*, *z*.
